# Seed Fatty Acids Modify Oviposition of *Tenebrio molitor* (Coleoptera: Tenebrionidae)

**DOI:** 10.3390/plants14060848

**Published:** 2025-03-08

**Authors:** Gabrielė Bumbulytė, Arijus Auškalnis, Vincas Būda

**Affiliations:** 1 Laboratory of Chemical and Behavioural Ecology, Nature Research Centre, Akademijos Str. 2, LT-08412 Vilnius, Lithuania; arijusaus@gmail.com (A.A.); vincas.buda@gamtc.lt (V.B.); 2 Life Sciences Center, Vilnius University, Saulėtekio al. 7, LT-10223 Vilnius, Lithuania

**Keywords:** inhibitor, linoleic, oleic acid, palmitic, repellent, stearic

## Abstract

Plant–insect interactions mediated by chemical compounds are well documented in roots and above-ground plant parts except seeds. The latter chemoecological interactions remain poorly studied. The chemical composition of seeds, including attractive, repellent, or inhibitory compounds, likely influences oviposition behavior, yet specific studies on this subject are scarce. This study evaluated the oviposition behavior of the yellow mealworm (*Tenebrio molitor* L. (Coleoptera, Tenebrionidae)) on substrates of common oat (*Avena sativa* L. (Poales: Poaceae)), common wheat (*Triticum aestivum* L. (Poales: Poaceae)), rapeseed (*Brassica napus* L. (Brassicales: Brassicaceae)), and pure sand. Females laid the most eggs on oat and wheat substrates, while oviposition on sand was reduced by 22%. The chemoreceptors located in the antennae of *T. molitor* were found not to influence oviposition. Hexane extracts of oat flour were found to contain oviposition-inhibiting compounds, identified as fatty acids. Behavioral tests showed that oleic, palmitic, linoleic, and stearic acids inhibited oviposition at concentrations ranging from 5% to 0.5%. A lower concentration (0.05%) did not have this effect. Additionally, linoleic, palmitic, and oleic acids exhibited repellent properties, whereas stearic acid did not. These findings provide valuable insights into optimizing substrate composition to enhance *T. molitor* reproduction. This has applications for small-scale laboratory research and large-scale industrial production, supporting the use of *T. molitor* as an alternative protein source for feed and food.

## 1. Introduction

Plant–insect interactions mediated by chemical compounds are well studied in roots and above-ground plant parts [1], playing critical roles in plant and insect life cycles. The exception is chemoecological interactions involving seeds. Those remain underexplored, despite their importance for fundamental understanding and practical applications.

Insects are well known for their significant roles in seed predation [2] and dispersal [3]. In rare cases, they even enhance seed germination. For example, the cotton seed bug, *Oxycarenus luctuosus* Montrouzier (Hemiptera: Lygaeidae), enhances *Gossypium* (Malvales: Malvaceae) seed germination by breaking impermeable seed coats [4]. Some plant seeds possess defensive chemical compounds, which can be either toxic or repellent to insects, deterring herbivory. A notable example is the common bean, *Phaseolus vulgaris* L. (Fabales: Fabaceae), which exhibits resistance to the cowpea seed beetle, *Callosobruchus maculatus* Fabricius (Coleoptera: Chrysomelidae), through seed coat repellency [5].

The mechanisms guiding insect seed recognition for oviposition or feeding remain poorly understood. Seed chemistry, including attractive, repellent, or inhibitory compounds, likely plays a critical role. For instance, ackee (*Blighia sapida* K.D.Koenig (Sapindales: Sapindaceae)) seed extracts reduce oviposition in multiple pests, but the active compounds remain unknown [6]. Similarly, repellency in common bean seeds against *C. maculatus* lacks identification [5].

Seeds of cultivated plants, along with their insect pests, offer suitable models for studying seed–insect interactions mediated by chemical compounds. In this study, we focused on common wheat, *Triticum aestivum* L. (Poales: Poaceae); common oat, *Avena sativa* L. (Poales: Poaceae); and rapeseed, *Brassica napus* L. (Brassicales: Brassicaceae), as seed models. The seed-feeding beetle *Tenebrio molitor* L. (Coleoptera, Tenebrionidae), commonly known as the yellow mealworm, was selected as the insect model due to its widespread occurrence as a stored-product pest and its economic importance globally. While *T. molitor* is primarily considered a pest of stored grains and grain products, it is omnivorous in its natural environment [7]. Furthermore, this species has recently gained attention for its potential as a source of environmentally sustainable protein and as an alternative feed and food product.

The objective of this research was to identify chemical compounds within the seeds of rapeseed, common wheat, and common oat that affect the oviposition behavior of the seed-feeding insect *T. molitor*. Such findings could provide valuable insights not only for fundamental ecological studies but also for optimizing insect rearing under laboratory and industrial conditions.

## 2. Results

### 2.1. Oviposition on Different Substrates

**Figure 1:**
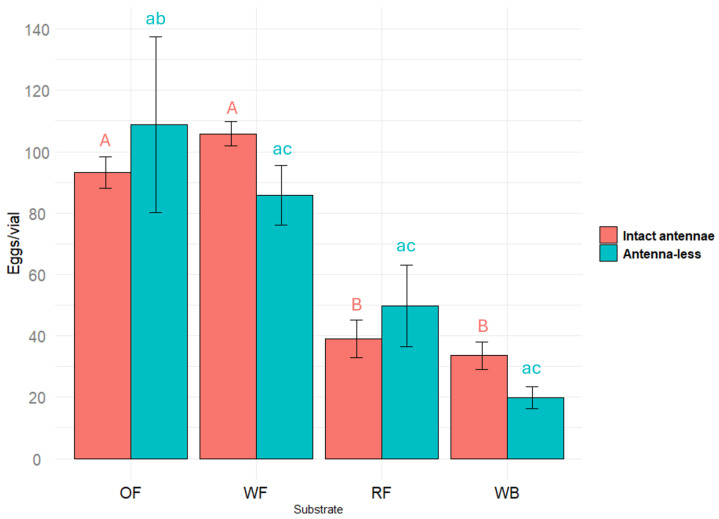
Number of eggs laid by *Tenebrio molitor* females with intact antennae and antenna-less females on different flour substrates: oats (OF), wheat (WF), rapeseed (RF), and wheat bran (WB). Results were analyzed using a one-way ANOVA followed by Tukey’s test. Significant differences are indicated by different letters (uppercase for results from females with intact antennae and lowercase for results from antenna-less females) at *p* < 0.05. Error bars represent the standard error.

The effect of different substrates (oat flour, wheat flour, rapeseed flour, and wheat bran) on *Tenebrio molitor* (with intact antennae) egg laying was significant (F(56.02) = 3, *p* < 0.001) (ANOVA). Females with intact antennae laid significantly more eggs (*p* < 0.05) on oat flour and wheat flour compared both to rapeseed flour and wheat bran (Figure 1).

### 2.2. The Substrate VOCs and Oviposition

The amputation of the antennae (i.e., the removal of the most abundant olfactory sensillae) did not cause a very pronounced effect in the oviposition test. But the effect of all different substrates (oat flour, wheat flour, rapeseed flour, and wheat bran) on antenna-less *Tenebrio molitor* egg laying was significant (F(5.601) = 3, *p* = 0.0229) (ANOVA).

In the antennae-less female sample, which was of the same size as that of females with intact antennae, the standard error increased (this could be a consequence of some damage caused during the antennaectomy). Due to the higher data scattering, the statistical estimates of the differences were less pronounced, but as in the first case, the lowest number of eggs was laid on the substrate of wheat bran, while substrates of oat and wheat flour remained in the group of most suitable for oviposition (Figure 1).

The results obtained indicate that the VOCs of the substrates perceived by olfactory sensillae localized in the antennae of *T. molitor* females did not modulate their oviposition behavior.

### 2.3. Oviposition on Sand

For the search for biologically active compounds that influence oviposition, it makes sense to have a control substrate without organic compounds but with other properties suitable for egg laying. We chose sand as a possible candidate for such a substrate. It is well known; its composition consists of minerals, with the main component being silicon dioxide (SiO_2_).

**Figure 2:**
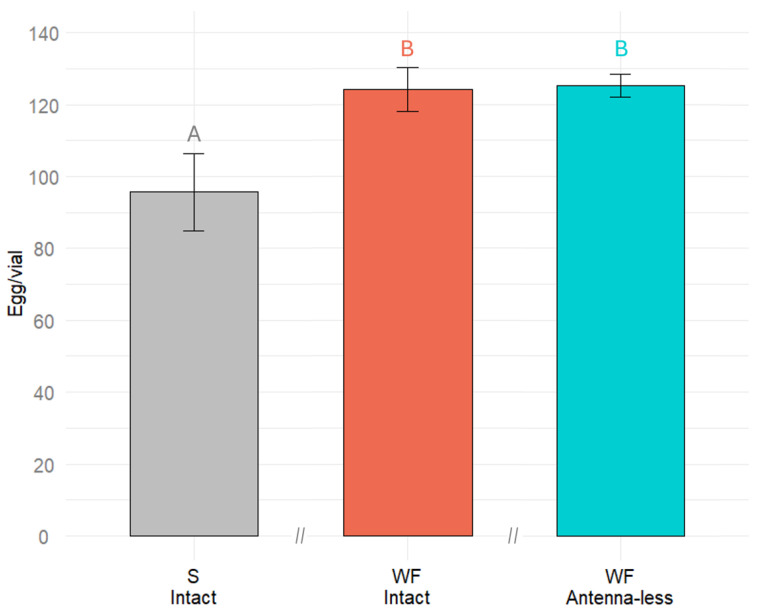
Number of eggs laid by *Tenebrio molitor* intact and antenna-less females to either sand (S) or wheat flour (WF) substrate. Results were analyzed using a one-way ANOVA followed by Tukey’s test, with significant differences indicated by different letters (*p* < 0.05). Error bars represent the standard error.

The effect of different substrates (wheat flour and sand) and antenna being (intact or antenna-less) on *Tenebrio molitor* egg laying was significant (F(5.203) = 3, *p* = 0.0236) (ANOVA). In the sand substrate, the females laid about 78% of the number of eggs they lay in their natural wheat flour substrate (Figure 2). A decrease of about 22% was statistically significant; however, it is obvious that this organic-free substrate still can be used for searching for chemical compounds affecting oviposition.

### 2.4. The Effect of Oat Extract

**Figure 3:**
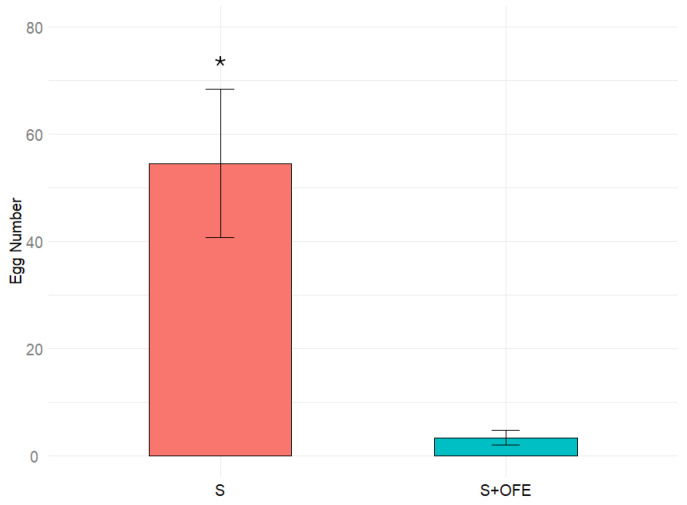
Number of eggs laid by *Tenebrio molitor* females on sand (S) and sand plus oat flour extract (S + OFE). Results were analyzed using a Wilcoxon test, and significant difference with *p* < 0.05 are indicated by asterisk (*). Error bars represent the standard error.

Data obtained by testing the effects of chemical compounds of oats on oviposition are given in Figure 3. On the sand with oat extract, the females laid just about 4% of the number of eggs compared to on the pure sand. This big difference was statistically significant (*p* > 0.05), and thus the result obtained clearly testifies to the inhibitory effect of the extract.

### 2.5. The Effect of Oat Extract Fractions

To reveal which compounds are present in the extract affect oviposition, fractionation of the extract (complex mixture of compounds) is necessary. We divided the extract into two fractions and tested their effects. For control, oviposition on the substrate of extracted oat was used. In the extract (a mixture consisting of many chemical compounds), some compounds can act as inhibitors and some as stimulators. In this case, the total effect of the mixture is determined by predominant compounds. Among such masked compounds could be both volatile and non-volatile compounds. Therefore, the extract fractions were tested on females both with intact and removed antennae.

**Figure 4:**
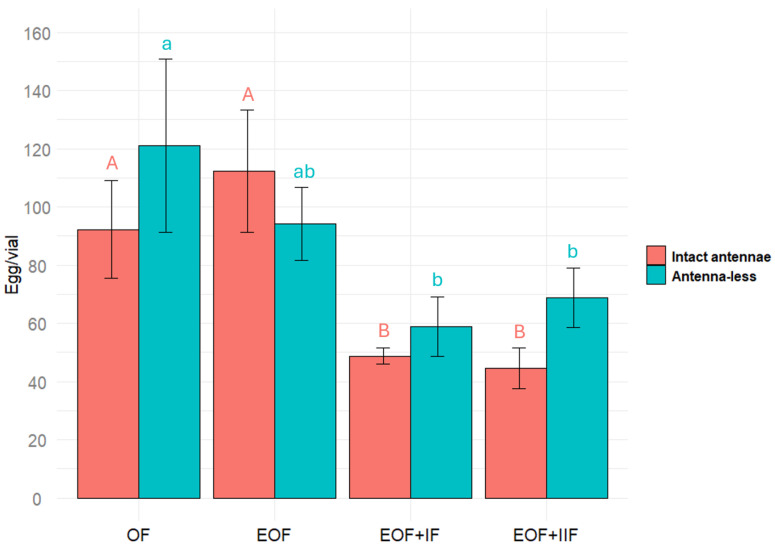
Number of eggs laid by *Tenebrio molitor* female on different substrates (oat flour (OF), extracted oat flour (EOF), extracted oat flour plus 1st fraction (EOF + IF), and extracted oat flour plus 2nd fraction (EOF + IIF)), both intact and antennae-less. Results were analyzed using a Friedman test followed by post hoc Wilcoxon signed-rank tests with Bonferroni correction, with significant differences indicated by different letters (*p* < 0.05). Error bars represent the standard error.

The effect of different substrates (oat flour, extracted oat flour, extracted oat flour plus 1st fraction, and extracted oat flour plus the 2nd fraction) on *Tenebrio molitor* (with intact antennae) egg laying was significant χ^2^(3) = 19.1, *p* < 0.001 (Friedman test), but the effect on antenna-less *T. molitor* was not significant χ^2^(3) = 7.4, *p* = 0.06018 (Friedman test). Females, with antennae, laid more eggs on extracted oat compared to the same substrate plus oat extract fraction (either the first or the second). This difference was statistically significant, *p* < 0.05 (Figure 4). The data obtained reveal that there may be more than one of the compounds that inhibit *T. molitor* female’s oviposition and that olfactory stimuli perceived by antennae can be involved in the process of inhibition of the egg-laying.

### 2.6. The Effect of Fatty Acids

Oat extract contained a noticeable layer of fat, so for the study of its biological effect, we looked at the composition, based on already known (published) data. Four of the most abundant fatty acids were selected: linoleic, oleic, palmitic, and stearic, each of which accounts for from 1 to 36% of the fatty acid content of oat grain [8].

In a two-choice test, *T. molitor* females were given the option to lay eggs on either plain sand or sand treated with a fatty acid. The number of eggs laid by the females varied significantly depending on the type of fatty acid and its concentration.

**Figure 5:**
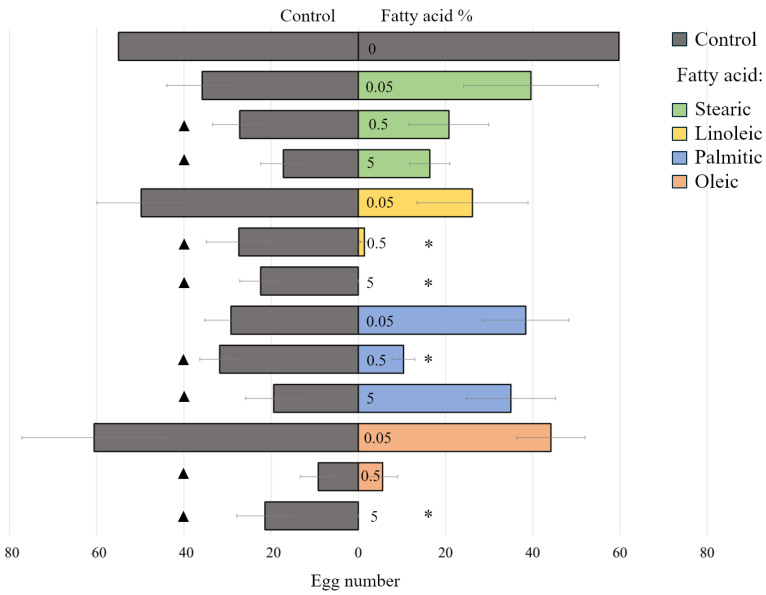
Oviposition of *Tenebrio molitor* females on substrates with four different fatty acids (linoleic, oleic, palmitic, and stearic) and control plates in two-choice test. Tested concentrations: 5; 0.5; and 0.05%. Statistically significant difference (*p* < 0.05) between egg counts on the stimulus and control plates within the same box was marked by asterisk (Wilcoxon test); the same significance in egg counts between the fatty acid test and control test was marked by triangles (one-way ANOVA, followed by Tukey’s test). Error bars represent the standard error.

The effect of different fatty acids (stearic, linoleic, palmitic, and oleic) on *T. molitor* egg laying was significant (F(5.68) = 12, *p* < 0.001) (ANOVA). When comparing the total number of eggs laid across both options (plain sand and fatty-acid-treated sand), it was found that fatty acids at both 5% and 0.5% concentrations significantly reduced egg-laying overall (Figure 5). This inhibitory effect was observed not only because females avoided laying eggs on the sand with fatty acid but also because they laid fewer eggs on the plain sand option as well (Figure 5). In contrast, the lowest tested concentration (0.05%) of any fatty acid had no such effect.

Besides their inhibitory effect, the repellent feature of linoleic, palmitic, and oleic acids was revealed: this was evidenced by the asymmetry of the egg distribution between the control and the test plates under free choice conditions. A statistically significant effect (*p* < 0.05) was caused by a 5% concentration of oleic and linoleic fatty acids and a concentration of 0.5% of linoleic and palmitic fatty acids (Figure 5).

Neither inhibitory nor repellent effects caused the lowest concentration (0.05%) of the fatty acids tested (Figure 5).

## 3. Discussion

While the effects of crate size, oviposition duration, adult density, and cannibalism on the reproductive performance of *T. molitor* have been well documented [9], there is a notable gap in understanding the impact of chemical substances in the feed substrate on oviposition. These include stimulatory, inhibitory, deterrent, and repellent effects caused by substrate compounds on the females of this species.

It is known that *T. molitor* females avoid poor-quality food when selecting oviposition sites and usually lay fewer eggs and die sooner when poor-quality food is present [10]. The present study demonstrated that even among high-quality substrates, females oviposit differently. Females exhibited a strong preference for certain substrates, laying the most eggs on oat flour, followed by wheat flour, and significantly fewer eggs on rapeseed flour and wheat bran. This finding highlights oat flour as one of the most favorable substrates for oviposition, likely due to its advantageous physical and chemical properties.

For some insect species, such as the European grape worm, *Lobesia botrana* Denis and Schiffermüller (Lepidoptera: Tortricidae) [11], and the oriental fruit fly, *Bactrocera dorsalis* Hendel (Diptera: Tephritidae) [12], olfactory stimuli are important for oviposition, while for others, like the tobacco hawk moth, *Manduca sexta* L., (Lepidoptera: Sphingidae) [13], they are not. In the case of *B. dorsalis*, it has been established that olfactory receptors associated with oviposition are localized in the antennae [12]. Data on egg-laying regulation in coleopterans, such as the spotted lady beetle, *Coleomegilla maculata* De Geer (Coleoptera: Coccinellidae) [14,15,16], are scarce, and the involvement of antennal chemoreceptors has not yet been analyzed.

The results of the present research clearly demonstrate that *T. molitor* females’ antennal chemoreceptors are not involved in the perception of chemical compounds influencing oviposition. The result of our study was obtained in a situation when the phase of the search for a substrate from a distance was eliminated (i.e., the phase when the olfaction and olfactory receptors, localized mostly on the antennae, would have been needed), as the insects and the substrates for oviposition were already at a close range (in small cages).

Before testing the role of chemical compounds in egg-laying, the suitability of a sand substrate that does not contain any organic compounds was assessed. It was found that *T. molitor* females lay their eggs to pure sand substrate, but with lower intensity (approximately 25% less) compared to wheat flour. For future studies, it would be beneficial to include a control group with antennae removed and ovipositing on the neutral/control substrate (sand) to better understand the role of antennal chemoreception in oviposition behavior.

Testing the sand substrate with an oat extract resulted in a significant inhibition of oviposition. This result indicates that chemical cues within the substrate play a crucial role in *T. molitor* females’ oviposition behavior. It suggests that the extract contains compounds that suppress oviposition.

The hexane oat extract, when left undisturbed, separated into two layers, with the upper layer undoubtedly consisting of fats. Since the chemical composition of oat fatty acids is known (as well as that of other plant seeds tested; see Table 1), we proceeded to test the most abundant components.

Each of the fatty acids tested (oleic, palmitic, linoleic, and stearic) at concentrations ranging from 5% to 0.5% inhibited oviposition in *T. molitor* females. A lower concentration (0.05%) did not have this effect. Very little is known about the impact of fatty acids on the oviposition of other insect species. In spotted wing drosophila, *Drosophila suzukii* Matsumura (Diptera: Drosophilidae), caprylic and capric acids were identified as key components responsible for oviposition deterrence, while the other fatty acids tested had no significant effect [25]. Fatty acids were also found to be important cues for oviposition in the black soldier fly, *Hermetia illucens* L. (Diptera: Stratiomyidae): decanoic acid is repulsive, while tetradecanoic acid is attractive to females and stimulates oviposition [26]. In *Ostrinia latipennis* Warren (Lepidoptera: Crambidae), fatty acids with C_9_–C_22_ stimulate oviposition [27]. A mixture of fatty acids stimulates oviposition in the cowpea weevil, *Callosobruchus maculatus*, but elevated levels of oleic acid act as a deterrent [28]. A blend of unsaturated fatty acids decreases oviposition in the pepper weevil, *Anthonomus eugenii* Cano (Coleoptera: Curculionidae) [29]. The effect of fatty acids on oviposition in the oriental fruit fly, *Bactrocera dorsalis*, was more complex: four fatty acids (caprylic, capric, oleic, and linoleic acids) reduced oviposition, two (lauric and myristic acids) had no effect, and two (palmitic and stearic acids) stimulated oviposition [30]. A similar complex effect was observed in another fruit fly species, the melon fly, *Zeugodacus cucurbitae* Coquillett (Diptera: Tephritidae) [31]. These examples demonstrate that the effect of fatty acids on oviposition is ambiguous and species-dependent, influenced by the organism’s biological peculiarities. To date, there have been no reports on the inhibitory effect of fatty acids on *T. molitor* beetles.

In addition to the inhibitory effects, repellent properties were observed for linoleic, palmitic, and oleic acids, while stearic acid did not exhibit this effect. The dual effects of the same fatty acid on the same organism are known [31], though such cases are extremely rare, to our knowledge.

Since both inhibitory and repellent effects were recorded in our tests at concentrations ranging from 5% to 0.5% (but not at 0.05%), a reasonable question arises about the relevance of these findings to real-world conditions. The answer can be found by reviewing previously published data on the qualitative and quantitative composition of fatty acids in grains, as presented in Table 1. These data suggest that *T. molitor* may encounter concentrations of oleic, palmitic, and linoleic fatty acids significantly higher than 5% in natural substrates. The results reported in this paper can help optimize media composition for *T. molitor* reproduction, both for small-scale laboratory research and large-scale industrial purposes. For the most successful reproduction of *T. molitor,* the medium within oviposition chambers must contain less than 5 percent of each of the following fatty acids: oleic, palmitic, and linoleic.

## 4. Materials and Methods

### 4.1. Insects

Yellow mealworms (*Tenebrio molitor* L.) were reared in the Laboratory of Chemical and Behavioural Ecology, Nature Research Centre Vilnius, Lithuania on a diet of wheat bran supplied with carrot pieces. To obtain virgin adults, pupae were sexed based on the morphological characteristics of the terminal segments. The pupae were allowed to undergo the final stages of metamorphosis in plastic containers, so that males and females hatched separately. Upon hatching, the adult insects were maintained for 7 days under controlled environmental conditions: a temperature of 25 °C, 60% relative humidity, and a 12:12 h light-dark cycle.

Before mating, male beetles were marked with a green dot (Acrylic marker SCHNEIDER Paint-it 310, 2 mm, green, Wernigerode, Hartz, Germany) on the dorsal side of the thorax to distinguish them from females. The beetles were allowed to mate for 5 days under the same controlled conditions as indicated above and were used in the tests. For olfactory blocking, the antennae were removed at the very base using surgical scissors.

### 4.2. Oviposition Substrates

Therefore, for biologically active compounds, we chose the following as substrates: oat flakes produced by “Dobele” (Latvia), wheat flour by “Kauno grūdai” (Lithuania), rapeseed flour by “EKKO” (Lithuania), and wheat bran by “Malsena” (Lithuania). Oat flakes were ground into flour and sieved through a 1 mm sieve. The quartz sand was 0.0–0.4 mm fraction produced by “Anykščių kvarcas” (Lithuania). Before use, the sand was washed and heated at 150 °C for 2 h. Solvent hexane was used (purchased from Honeywell, Seelze, Offenbach, Germany, purity ≥ 99%).

### 4.3. Fatty Acids

All fatty acids were bought from Thermo Fisher Scientific (Vilnius, Lithuania) and were of analytical grade: oleic acid (≥98.5% purity, GC), linoleic acid (≥98.5% purity, GC), palmitic acid (≥97.5% purity, GC), and stearic acid (≥97.5% purity, GC).

### 4.4. Oviposition Test

During the test, there was a box (36 × 26 × 11 cm) containing a mesh-bottomed inner box (35 × 24 × 10 cm; mesh size 1 mm). Beneath the mesh were plastic Petri dishes (3 cm diameter) with different substrates (20 g) for female oviposition (except in sand substrate test).

A total of 10 male and 10 female (intact or antennae-less) beetles were released onto a mesh-bottomed box. A 10 g piece of carrot was added as water source. It was maintained at 25 °C and 60% relative humidity with a 12:12 h light-dark cycle. After 24 h, the substrates from the dishes were sieved, and the eggs were counted. Each test was replicated five times for each substrate.

#### 4.4.1. Difference Between Substrates

For the four-choice test, a Petri dish was supplied with each substrate (oat, wheat, rapeseed flour, and wheat bran). Used female beetles were intact or antennae-less.

#### 4.4.2. Sand Substrate

Beneath the mesh of each inner box, 100 g of one of two different substrates (sand or wheat flour) were placed. Used female beetles were intact or antenna-less.

#### 4.4.3. Oat Extract

For the test, 150 mL of hexane was added to 100 g of ground oats. The mixture was shaken and sonicated in an ultrasonic bath for 480 s, followed by a 1-h extraction period. The settled extract was collected and poured on to 100 g of sand. Beneath the mesh, two Petri dishes were positioned with sand plus oat extract and sand with evaporated solvent.

#### 4.4.4. Oat Extract Fractions

For the test, 150 mL of hexane was added to 100 g of oats in a tightly sealed container. The mixture was thoroughly shaken and sonicated in an ultrasonic bath for 480 s, followed by a 1-h extraction period. The settled extract was then collected and kept in fridge (+5 °C) for 1 h. After this time, the first and second fractions were collected and poured on extracted oat flour. In each four-choice test box, four dishes containing oat flour, extracted oat flour, extracted oat flour plus the first fraction, and extracted oat flour plus the second fraction were placed.

#### 4.4.5. Fatty Acid Analysis

For the test, 30 mL of hexane was poured onto 200 g of the sand, thoroughly mixed, and the solvent was allowed to evaporate. In two control dishes, hexane-treated sand was placed.

Solutions of fatty acids in hexane were prepared by weighing 1 g (5%), 0.1 (0.5%) g, and 0.01 (0.05%) g of each fatty acid and placing them into separate, tightly sealed vials. To each vial, 15 mL of hexane was added, and the mixture was thoroughly shaken. The solutions were sonicated in an ultrasonic bath for 480 s. Each solution was individually poured onto 100 g sand. The solvent was evaporated by mixing until dry mass.

Beneath the mesh, two dishes were positioned: one experimental dish containing the fatty acid-treated sand and one control dish with the solvent-treated sand.

### 4.5. Statistical Analysis

Statistical analysis of the data was conducted using Microsoft Excel, R version RStudio 2024.09.1+394 (R Core Team, 2023) within the RStudio environment (RStudio Team, 2023). For the analysis of oviposition on different substrates, and oviposition on sand, a one-way ANOVA was applied followed by Tukey’s HSD to determine significant differences between groups. For oviposition in the presence of oat extract fractions, the Friedman test was applied followed by post hoc Wilcoxon signed-rank tests with Bonferroni correction to determine significant differences between groups. For oviposition in the presence of oat extract, the Wilcoxon matched-pair test, and for fatty acid analysis the Wilcoxon matched-pair test and Mann–Whitney U test, were employed. Statistical significance was determined at *p <* 0.05, indicating a confidence level higher than 95%.

## Figures and Tables

**Table 1:** Fat content and fatty acid composition of substrates (oat, wheat, rapeseed, and wheat bran).

		Fatty Acids %			
Substrate	Fat Amount %	Oleic	Palmitic	Linoleic	Stearic
Oat [8,17,18,19]	4–10	34.80	18.90	38.30	2.10
Wheat [20]	2–4	11.80	21.10	58.30	3.10
Rapeseed [21,22]	40–45	62.25	4.70	17.19	1.80
Wheat bran [23,24]	3–4	14.60	17.70	54.60	2.60

Kouřimská et al., 2018 [8]; Sterna et al., 2014 [17]; Leonova et al., 2008 [18]; Sahasrabudhe, 1979 [19]; Lidon et al., 2019 [20]; Chew, 2020 [21]; Waheed et al., 2013 [22]; Apprich et al., 2014 [23]; and Jung et al., 2010 [24].

## Data Availability

The data presented in this study are available in the article.
